# Inter- and intra-year variation in foraging areas of breeding kittiwakes (*Rissa tridactyla*)

**DOI:** 10.1007/s00227-014-2477-8

**Published:** 2014-06-26

**Authors:** G. S. Robertson, M. Bolton, W. J. Grecian, P. Monaghan

**Affiliations:** 1 Institute of Biodiversity, Animal Health and Comparative Medicine, Graham Kerr Building, University of Glasgow, Glasgow, G12 8QQ UK; 2 RSPB Centre for Conservation Science, The Lodge, Sandy, Bedfordshire, SG19 2DL UK

## Abstract

While seabird conservation efforts have largely focused on protection from threats at the colony (e.g. reducing disturbance and predation), attention is increasingly being given to implementing protection measures for foraging areas at sea. For this to be effective, important foraging areas must be identified. Although numerous studies have examined seabird foraging behaviour, information is still lacking on the variability in area utilisation within and among breeding seasons. GPS devices were attached to adult black-legged kittiwakes breeding at an expanding North Sea colony (55°20′N, 1°32′W) during both incubation and chick-rearing in 2012 and during chick-rearing in 2011, to determine whether foraging areas remained consistent and to identify the oceanographic characteristics of areas used for foraging. The type and size of prey items consumed at different stages of the breeding cycle was also examined. During incubation (April–May 2012), kittiwakes foraged substantially further from the colony and fed on larger sandeels than when feeding chicks, and there was significant inter-annual variation in foraging areas used during the chick-rearing period (June–July 2011 and 2012). Foraging areas were characterised by cooler sea surface temperatures and areas of high chlorophyll *a* concentration, although association with specific oceanographic features changed within the breeding season and between years. These results emphasise the importance of considering how foraging areas and reliance on specific oceanographic conditions change over time when seeking to identify important marine areas for seabirds.

## Introduction

Black-legged kittiwakes (*Rissa tridactyla*, hereafter “kittiwake”) are small surface-feeding seabirds widely distributed in temperate and Arctic regions in the Northern Hemisphere (Harrison 1983). Historically, kittiwakes have bred in large numbers along the north-western edge of the North Sea (Lloyd et al. 1991), but have recently become a species of conservation concern as their abundance and productivity in the North Sea have declined in the last 30 years (Harris and Wanless 1990, 1997; Wanless and Harris 1992; Upton et al. 2000; Mitchell et al. 2004; Eaton et al. 2009). An important factor contributing to this decline is a reduction in food availability due to decreases in the abundance of principal forage fish such as lesser sandeel (*Ammodytes marinus*, hereafter “sandeel”) (Harris and Wanless 1990; Rindorf et al. 2000; Daunt et al. 2002; Frederiksen et al. 2004, 2008). Kittiwakes are obligate surface-feeders restricted to obtaining food from the top few metres of the water column (Harris and Wanless 1990; Coulson 2011). Previous studies have identified surface-feeding seabird species as being more severely affected by food shortages than diving species (Furness and Ainley 1984; Furness and Tasker 2000). Kittiwakes are especially vulnerable to reductions in prey abundance as they have high foraging costs, restricted diving ability and limited ability to switch to different prey types (Furness and Tasker 2000).

It is becoming increasingly apparent that protection of seabird foraging areas is necessary to mitigate threats caused by human activities at sea such as marine developments, overfishing, fishery bycatch of seabirds and pollution (Monaghan 1996; Lewison and Crowder 2003; Garthe and Hüppop 2004; Votier et al. 2005; Scott et al. 2006; Grecian et al. 2010). Marine protected areas (MPAs) are a useful conservation measure to reduce threats to marine life by limiting human activities in important foraging areas (IUCN 1988). For many seabird species data describing the use of offshore areas are limited, making identification of suitable MPAs difficult (Lewison et al. 2012). In recent years, bird-borne GPS devices have been successfully employed to identify foraging areas of a variety of species (Wood et al. 2000; Ryan et al. 2004; Weimerskirch et al. 2005; Kotzerka et al. 2010; Stauss et al. 2012).

Kittiwakes are useful species in which to examine variation in foraging behaviour for several reasons. Firstly, their foraging areas have been shown to vary depending on environmental conditions and food abundance (Suryan et al. 2000; Scott et al. 2010). Secondly, kittiwake populations have been shown to fluctuate in synchrony with sandeel abundance (Frederiksen et al. 2004) and are therefore good indicators of the health of the marine environment (Parsons et al. 2008). Thirdly, understanding foraging distributions of kittiwake colonies in the North Sea after the closure of the sandeel fishery in the Wee Bankie in 2000 demonstrates the effectiveness of offshore foraging area protection (Daunt et al. 2008). The recent miniaturisation of data loggers has allowed total duration of kittiwake foraging trips to be recorded and important foraging areas to be identified (Kotzerka et al. 2010; Chivers et al. 2013; Redfern and Bevan 2014).

Despite the large number of tracking studies carried out to date, most have considered only a single breeding phase (Lewis et al. 2002; Weimerskirch et al. 2007; Stauss et al. 2012; Chivers et al. 2013) or breeding season (Weimerskirch et al. 2005; Kotzerka et al. 2010; Votier et al. 2010). As such, few studies have examined spatiotemporal shifts in foraging behaviour at different stages of the breeding cycle, or in different years (Weimerskirch et al. 1993; Hull et al. 1997; Berrow et al. 2000; Stauss et al. 2012; Chivers et al. 2013). Local prey distribution and abundance is strongly influenced by oceanographic conditions; therefore, foraging areas used during breeding are likely to change through time (Monaghan et al. 1994; Suryan et al. 2002; Pinaud et al. 2005; Weimerskirch 2007; Chivers et al. 2013). Furthermore, the suitability of different areas is likely to be influenced by other factors such as the costs incurred by being away from the nest or variation in optimal prey size at different stages of the breeding cycle. Designating protected areas based on data collected only during 1 year or breeding stage may underestimate the size of foraging areas that need to be protected, but we lack information on variation in area use. Tracking studies carried out over longer temporal periods will improve our understanding of how foraging areas change over time and whether oceanographic conditions facilitate these changes. Such studies will improve our ability to make predictions regarding the distribution of seabirds at sea.

We examined the foraging behaviour of breeding kittiwakes at a North Sea colony at different stages of the breeding season in the same year and at the same breeding stage (chick-rearing) in two different years. We investigated (1) whether the location of foraging areas or adult condition during chick-rearing varied between the 2 years, (2) whether prey size, foraging area or adult condition varied with stage of the breeding cycle in the same year and (3) how changes in foraging areas related to variation in specific oceanographic conditions. We discuss the implications of our results for the identification of offshore protected areas for seabirds.

## Materials and methods

### Study site

The study took place on Coquet Island, Northeast England (55º20′N, 1º32′W) during chick-rearing from June to July 2011 and during incubation and chick-rearing from May to July 2012. Coquet Island is a small (5 ha) low-lying island, 2 km from the mainland coast. Kittiwakes started visiting Coquet in significant numbers in 1990, and a breeding colony was established in 1991 (Coulson and Coulson 2008). Since then the colony has expanded each year to 215 pairs in 2012.

### GPS tagging

Tags were deployed on a total of 30 birds in 2012, 7 of which were not recaptured, and 15 birds during chick-rearing in 2011, 2 of which were not recaptured. Hence, we retrieved movement data from adults in 13 nests during chick-rearing in 2011, 10 nests during incubation in 2012 and 13 nests during chick-rearing in 2012. One adult per nest was captured using a pole and noose under a permit issued by the British Trust for Ornithology. Each tagged bird was captured twice: once to deploy the tag and a second time to retrieve the tag and download the data. Some tags could not be retrieved as we were occasionally unable to recapture birds after deploying tags. No eggs were damaged from deploying or recovering tags during incubation. We ensured that the same nests were not used to capture adults more than once during the study. Body mass and head and bill length were recorded and captured birds were ringed and fitted with GPS tags (Mobile Action Technology GT120, rehoused in heat-shrink tubing), which weighed ≤14 g, less than 4 % of birds’ body mass (Caccamise and Hedin 1985; Hill and Robertson 1987). Tags were attached to the back feathers using thin strips of cloth-backed (TESA^®^) tape. Birds were processed and tagged within 20 min of capture. All flew normally after release and most returned to the nest within 10–15 min. GPS tags were programmed to acquire a position every 100 s and tests indicated they had an accuracy of approximately 20 m when birds were moving. Tags were removed ~2–4 days after deployment. Breeding success of birds fitted with GPS tags and a random sample of untagged control birds breeding on the same cliffs were compared in 2012 to determine whether there were any detectable effects of tag deployment on breeding performance. Breeding success of tagged birds was not recorded in 2011 due to conflicts with other studies taking place at the study site. Coquet Island is a highly sensitive conservation area supporting ~80 pairs of endangered roseate terns (*Sterna dougallii*) (Mitchell et al. 2004). In order to visit the kittiwake colony, researchers had to move through the tern colonies, which in 2011 were being studied intensively. Hence, to mitigate disturbance to sensitive species, regular visits to the kittiwake colony were limited.

Tagging during incubation took place from 23rd May–26th May 2012. During chick-rearing, birds were tagged from 14th June–17th June 2011 and 17th June–3rd July 2012. The tracking period during chick-rearing in 2012 was longer than that in 2011 as tagging had to be temporarily suspended from 19th June–25th June 2012 due to poor weather conditions. The difference in tracking start dates between years was caused by a difference in average laying dates at study colonies in 2011 and 2012; dates of first hatching were 31st May 2011 and 5th June 2012. We considered it necessary to ensure that the birds we tracked were feeding chicks of similar ages, since this was likely to be more important than the slight variation in tracking dates between years. Approximate chick age at nests where each adult was tracked was estimated using date of first hatching recorded from a subset of 112 nests in the centre of the kittiwake colony close to nests which were selected for tagging in both years. Estimates of dates of first hatching and dates on which tags were deployed were used to calculate chick age of tagged nests and were compared between years. Dates of first hatching were similar between tagged nests and the subset of 112 nests used to estimate first hatching dates in 2012 (5th June and 6th June), hence date of first hatching from the subset of nests is likely to provide a useful estimation of date of first hatching for tagged nests in both years. Chicks of tagged birds were likely to be similar ages in 2011 and 2012, as the estimated age of chicks in the study colony were 15.5 ± 0.65 and 15.2 ± 1.16 days old when tracking started in 2011 and 2012, respectively.

We found no evidence that fitting GPS tags affected breeding performance of kittiwakes, which we examined in 2012. Number of fledged chicks from nests where one adult was tagged was not significantly different from that of a random sample of 30 untagged pairs breeding on the same cliffs (1.50 ± 0.14, *N* = 30 and 1.13 ± 0.16, *N* = 30, respectively; GLM with Poisson error structure: χ_1_^2^ = 1.25, *P* = 0.26, *N* = 60). Despite being unable to determine the effect of deploying this kind of tag on kittiwakes in 2011 due to lack of data on breeding success of tagged pairs, previous studies have shown that GPS tags of similar weight and method of attachment had no detrimental effect on kittiwake reproductive performance (Kotzerka et al. 2010; Chivers et al. 2012).

### Prey type and adult body condition

In order to obtain information on the main prey type and size utilised during the tracking period, we collected spontaneous regurgitate samples from both adults and chicks while fitting and retrieving GPS tags during incubation and chick-rearing in 2012. Samples were stored in individual plastic containers. A saturated solution of biological washing powder (Biotex^®^) was added to each sample, and the container was left at approximately 20 °C for 3–5 days until all the flesh and soft material had been dissolved. This material was then filtered from the solution leaving only bones (Lewis et al. 2001a; Bull et al. 2004). We identified species composition and estimated fish length from vertebrae. Bones were identified to the lowest taxa possible using a binocular microscope (for small bones × 60 magnification and for large bones × 12 magnification) and keys in Watt et al. (1997). Anterior caudal bones in each sample were identified, the total horizontal length of bones was measured using a calibrated eye piece graticule (x12 magnification) and the corresponding fish length was estimated using regression equations in Watt et al. (1997). To examine variation in adult kittiwake body condition in relation to breeding stage and year, an index (g mm^−1^) was calculated by dividing body mass (g) by head and bill length (mm) (Chastel et al. 1995; Brinkhof 1997; Mateo et al. 1998; Whitfield et al. 1999; Weimerskirch et al. 2005).

### Environmental Variables

To characterise the marine environment around the colony and examine how oceanographic features relate to foraging areas, we extracted 4 km^2^ resolution monthly composites of remotely sensed sea surface temperature (SST °C) and chlorophyll *a* concentration (mg m^−3^) from the MODIS instrument onboard the Aqua (EOS PM) satellite (http://oceancolor.gsfc.nasa.gov/), and 30 × 30 arc second resolution bathymetry data (m) from the GEBCO_08 data set available from NERC Earth Observation Data Acquisition and Analysis Service (NEODAAS). We used night-time SST data (11 µl) to reduce any bias in daytime estimates due to solar heating. Previous studies have found that SST, chlorophyll *a* concentration and bathymetry correlate with prey distribution and abundance both during the breeding season and during the preceding winter (Lutjeharms 1985; Schneider 1997; Park et al. 2002; Weimerskirch et al. 2004; Pinaud et al. 2005; Hyrenbach et al. 2007). In particular, SST in winter has been found to affect the distribution and abundance of sandeels, known to be an important kittiwake prey species (Arnott and Ruxton 2002). We also extracted SST and chlorophyll *a* concentration 1 month before tracking took place to account for potential lag in relationships between these variables and prey abundance. We retrieved mean monthly composites of SST and chlorophyll *a* concentration from concurrent months (May–July 2012 and June 2011 (lag 0)) and from 1 month previous to tracking commencing (April–June 2012 and May 2011 (lag 1)) as well as from the preceding winters (December–February 2012 and 2011) for use in environmental models.

### Data Analyses

Although a previous study on kittiwake foraging behaviour defined foraging trips as starting 300 m from the colony (Kotzerka et al. 2010), we increased this distance to 1 km in our study to exclude birds observed resting on rocks up to 1 km from Coquet Island (GS Robertson pers obs). We therefore classified behaviour at locations within 1 km of the colony or over land as maintenance, resting and nest attendance rather than foraging activities; a trip during which foraging may occur was defined as seaward movement beyond 1 km from the colony. Frequency distributions of flight speeds during incubation and chick-rearing in both years showed slight bimodality at speeds below 1 ms^−1^ and between 9 and 11 ms^−1^. As kittiwakes reduce their flight speed to collect food from the sea surface (Coulson 2011), we used periods of reduced flight speeds as indicators of foraging activity (Weimerskirch et al. 2004; Kotzerka et al. 2010). Birds were judged to be engaged in foraging behaviour at locations where instantaneous speed was <1 ms^−1^. This classification rule cannot discriminate between situations where birds foraged on the sea surface and where they rested between foraging bouts. Kittiwakes are known to rest on the sea surface while collecting food (Cramp and Simmons 1983; Coulson 2011). Without the use of saltwater or stomach temperature switches that record when birds were feeding, we could not definitively separate foraging and resting locations (these devices could not be attached to birds in our study due to weight restrictions (Wilson et al. 1995; Benvenuti and Dall’Antonia 2004)). However, this is unlikely to result in misrepresentation of foraging locations as surface-feeders such as kittiwakes forage primarily during daylight hours (Galbraith 1983; Weimerskirch and Guionnet 2002; Humphreys et al. 2007; Phalan et al. 2007) and locations where birds moved at speeds of <1 ms^−1^ during hours of darkness were removed before carrying out kernel density estimations and examining the effect of environmental variables on foraging, as these locations were likely to be where birds rested during the night.

For each foraging trip, we calculated maximum foraging range (most distant point from the colony (km)), total distance travelled (km) and trip duration (hr) and examined differences between trip parameters at both breeding stages and in different years using linear mixed models (LMMs) with bird ID as a random factor. Separate models were used to examine how time of day trips were carried out (day or night) affected variation in trip parameters during different breeding stages and years. Four individuals tracked during chick-rearing in both years and one individual tracked during incubation 2012 were excluded from models as their trips included <5 daylight foraging locations. These birds undertook trips with significantly smaller mean maximum foraging ranges (Welch’s *t* test: *t*_*11.28*_ = −2.71, *P* = 0.02, *N* = 36) than those of non-excluded individuals, although trip durations were not significantly different (*t*_*5.11*_ = 0.58, *P* = 0.58). Trips may have been carried out for purposes other than foraging, such as bathing or resting. Number of individuals included in further analyses from each breeding stage and year were as follows: Incubation in 2012 = 9 birds; Chick-rearing in 2012 = 12 birds and Chick-rearing in 2011 = 10 birds. Minimum adequate models were tested for normality by examining residual plots and response variables log-transformed where residuals showed heteroscedasticity. No recorded trips were incomplete.

We examined differences in foraging ranges (95 % volume contour) and core foraging areas (25 % volume contour) at different breeding stages and in different years using fixed kernel density estimation in a European Albers equal-area conic projection with a smoothing parameter (*h*) of 2.5 km and a grid size of 1 km^2^ (Suryan 2006). Kernel density plots were calculated in R version 2.15.2 using the adehabitatHR and maptools packages (Calenge 2006). Examination of possible breeding stage and year effects was carried out by quantifying overlap in foraging ranges and core foraging areas between incubation and chick-rearing in 2012 and during chick-rearing in 2011 and 2012. Percentage overlap was calculated by dividing the area of overlap between years/stages by the combined area utilised by foraging birds in both years/stages and multiplying by 100. This quantifies the degree of similarity between foraging areas used in different years and breeding stages. The percentage area of foraging ranges and core foraging areas found within those of another stage or year was also calculated. We used an area saturation curve method (Soanes et al. 2013) to determine whether foraging ranges and core foraging areas calculated for each breeding stage and year were likely to be representative of areas used by the whole colony.

Separate binomial generalised linear mixed models (GLMMs) were used to examine how foraging range changed at different stages of the breeding cycle and to identify environmental correlates of foraging locations. GLMMs were also used to examine how the distance that birds foraged from the colony changed during chick-rearing in two different years (2011 and 2012), whether changes could have been caused by the small difference in the timing of tracking studies within and between the 2 years and whether birds foraged in areas with similar environmental conditions in both years. To reduce interdependency among points, for each model presence data were 5 randomly selected foraging locations per track, and for absence data, 5 non-foraging locations per individual were randomly selected from a buffer zone around the colony (size of the buffer zone was defined as the maximum foraging range of all tracks in each breeding stage and in each year).

For models examining how environmental variables affected foraging locations, we fitted SST (lag 0), chlorophyll *a* concentration (lag 0), bathymetry, SST 1 month previously (lag 1), chlorophyll *a* concentration 1 month previously (lag 1), SST and chlorophyll *a* concentration the previous winter and their interactions as fixed effects and included bird ID as a random factor. Only uncorrelated fixed effects were included in the models (using Pearson’s product moment correlation coefficient where *r* ≥ 0.7 was taken to be a significant correlation). We first fitted the fully parameterised models using maximum likelihood (ML) and then removed terms by sequential deletion while testing for significant changes in model variance using likelihood ratio tests (LRTs) and by examining changes in AIC (Crawley 2007). We then refitted the minimum adequate model using restricted maximum likelihood (REML) to estimate effect sizes. Models were tested for goodness-of-fit using receiver operating characteristic (ROC) curves and the associated area under the curve (AUC). We used a LMM to test for differences in fish length between breeding stages in 2012 with bird ID as a random factor. The body condition indices of adults at different breeding stages and in different years were compared using Welch’s t tests. Analyses were carried out in R version 2.15.2 (R Development Core Team 2012) and ArcGIS version 10.1 (ESRI, USA). Means are presented ± 1 standard error throughout.

## Results

### Foraging areas

We obtained data from 106 foraging trips in total (Incubation *N* = 15, Chick-rearing in 2012 *N* = 60, Chick-rearing in 2011 *N* = 31). The amount of time GPS tags were attached to birds did not differ significantly between incubation and chick-rearing in 2012 (Welch’s t test: *t*_*19.35*_ = −0.76, *P* = 0.46, *N* = 22) or between chick-rearing in 2011 and 2012 (Welch’s t test: *t*_*18.24*_ = −0.10, *P* = 0.92, *N* = 25), and the percentage of tags retrieved was also similar between stages and years (Incubation 2012 = 76.92 %, Chick-rearing in 2012 = 76.47 %, Chick-rearing in 2011 = 86.67 %).

**Fig. 1:**
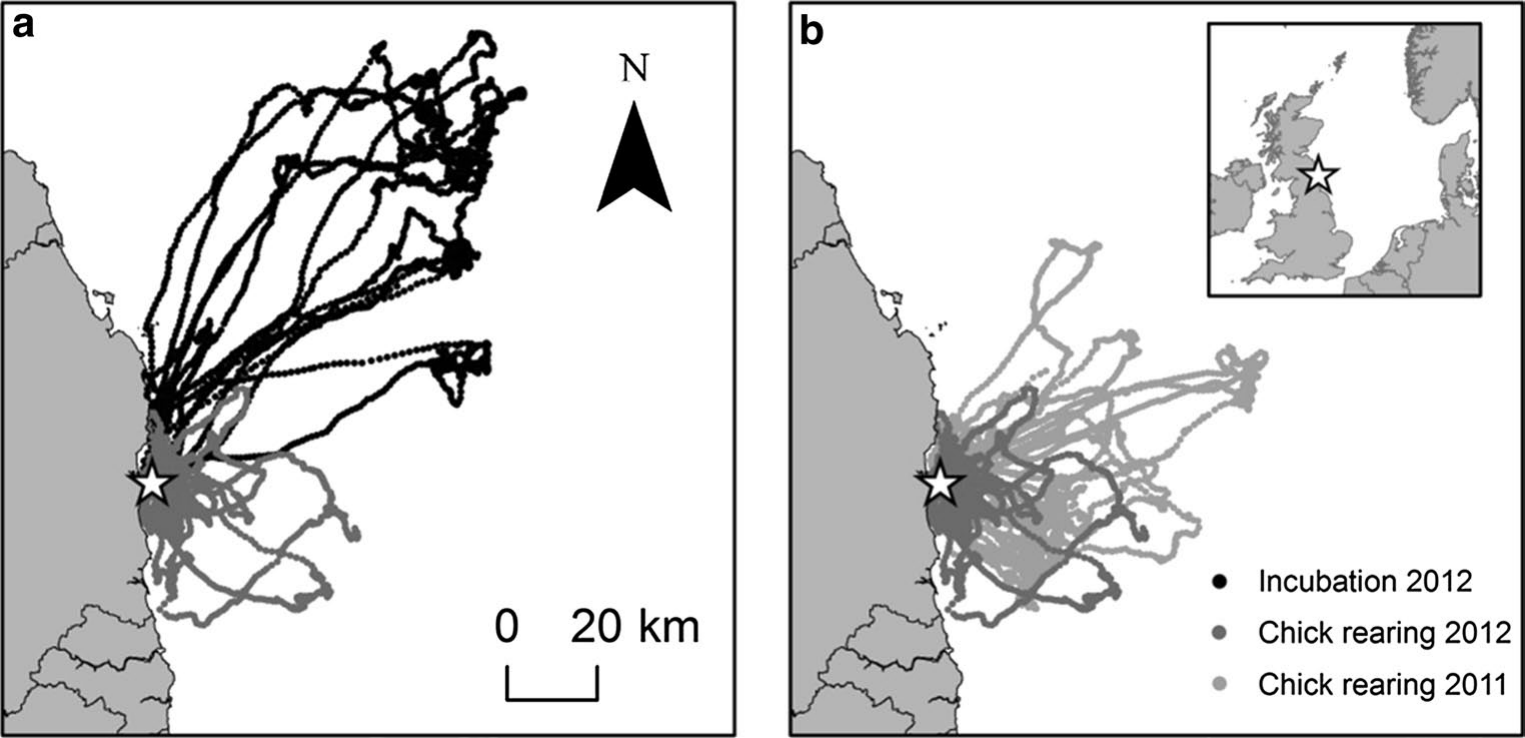
Kittiwake foraging tracks during **a** Incubation and Chick-rearing in 2012 and **b** Chick-rearing in 2011 and 2012 recorded from 23rd May to 3rd July 2012 and 14th June to 17th June 2011. Coquet Island (55°20' N, 1°32' W) is represented by a *star*

In 2012, birds foraged closer to the colony during chick-rearing than during incubation (Fig. 1, Table 1); maximum foraging range, total distance travelled and trip duration were all significantly greater during incubation. Despite the longer tracking period and maximum foraging range, total distance travelled and trip duration were significantly smaller during chick-rearing in 2012 compared with chick-rearing in the previous year (Fig. 1, Table 1). Models examining the effect of time of day on variation in trip parameters found significant interactions between breeding stage and time of day for all three trip parameters (χ_1_^2^ = 41.78, *P* < 0.001, *N* = 75; χ_1_^2^ = 33.22, *P* < 0.001 and χ_1_^2^ = 34.62, *P* < 0.001, respectively) ,which suggests that birds travelled further and for longer at night during incubation than during chick-rearing in 2012. Models for chick-rearing in 2011 and 2012 showed that time of day had a significant effect on maximum foraging range (χ_1_^2^ = 7.72, *P* < 0.001, *N* = 91) and trip duration (χ_1_^2^ = 31.93, *P* < 0.001), but no effect on total distance travelled (χ_1_^2^ = 0.10, *P* = 0.75). There were no significant interactions between year and time of day for maximum foraging range, total distance travelled and trip duration (χ_1_^2^ = 0.75, *P* = 0.39, *N* = 91; χ_1_^2^ = 0.10, *P* = 0.75; χ_1_^2^ = 1.45, *P* = 0.23, respectively).

**Table 1:** Table comparing trip parameters at different breeding stages and in different years

	Incubation (*N* = 15)	Chick-rearing (*N* = 60)	LRTs
Incubation and Chick-rearing 2012			
Max foraging range (km)	50.95 ± 12.99 (1.16–122.55)	9.03 ± 1.17 (2.21–47.55)	χ_1_^2^ = 12.99, *P* < 0.001
Total distance travelled (km)	129.62 ± 34.44 (0.22–324.84)	20.28 ± 3.24 (1.51–153.45)	χ_1_^2^ = 4.90, *P* = 0.03
Trip duration (h)	10.20 ± 2.55 (0.08–25.78)	2.87 ± 0.53 (0.36–30.20)	χ_1_^2^ = 16.38, *P* < 0.001
Chick-rearing 2011 and 2012			
	Chick-rearing 2011 (*N* = 31)	Chick-rearing 2012 (*N* = 60)	
Max foraging range (km)	28.02 ± 3.88 (1.15–77.63)	9.03 ± 1.17 (2.21–47.55)	χ_1_^2^ = 17.85, *P* < 0.001
Total distance travelled (km)	64.43 ± 9.19 (0.05–182.60)	20.28 ± 3.24 (1.51–153.45)	χ_1_^2^ = 9.44, *P* = 0.002
Trip duration (h)	5.07 ± 0.75 (0.08–14.12)	2.87 ± 0.53 (0.36–30.20)	χ_1_^2^ = 4.46, *P* = 0.03

Mean values are shown ± standard error with range given in brackets. Displays results of likelihood ratio tests (LRTs) from linear mixed models where response variables were log-transformed for models examining differences in max foraging range and total distance travelled between stages/years and where random factor = Bird ID. Incubation and Chick-rearing 2012 *N* = 75, Chick-rearing 2011 and 2012 *N* = 91

**Fig. 2:**
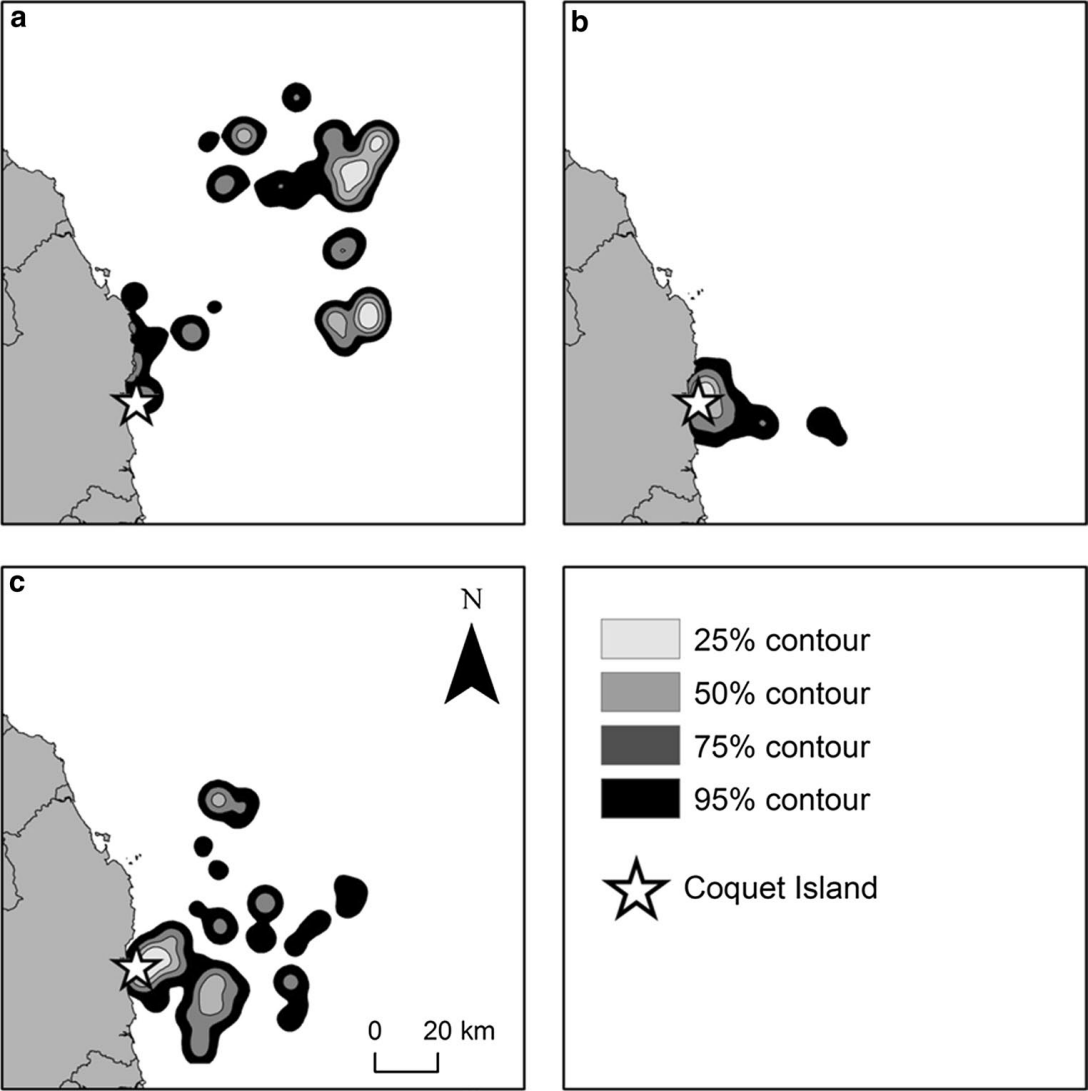
Kernel utilisation distribution of 3469 foraging locations (Incubation 2012 *N* = 964 locations; Chick-rearing 2012 *N* = 1539 locations; Chick-rearing 2011 *N* = 966 locations) using tracks from **a** 9 incubating birds in 2012, **b** 12 chick-rearing birds in 2012 and **c** 10 chick-rearing birds in 2011 foraging off Coquet Island. Contour plots show the density of locations on a 1 km^2^ grid using a 2.5 km smoothing parameter (*h*)

**Fig. 3:**
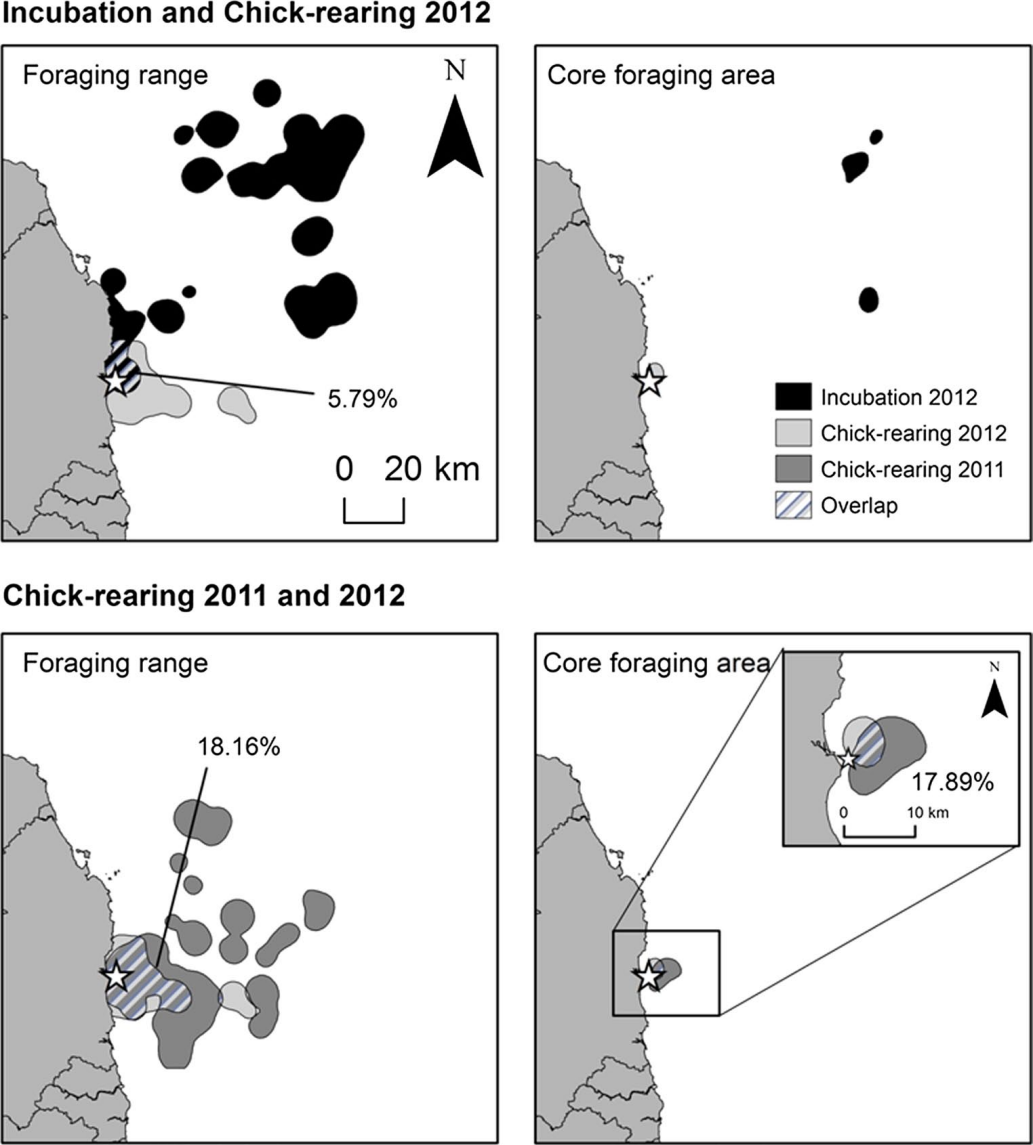
Percentage overlap between foraging ranges (95 % volume contour) and core foraging areas (25 % volume contour) during incubation and chick-rearing in 2012 and during chick-rearing in 2011 and 2012 calculated used a smoothing parameter of 2.5 km and a grid size of 1 km^2^. Coquet Island is represented by a star

**Fig. 4:**
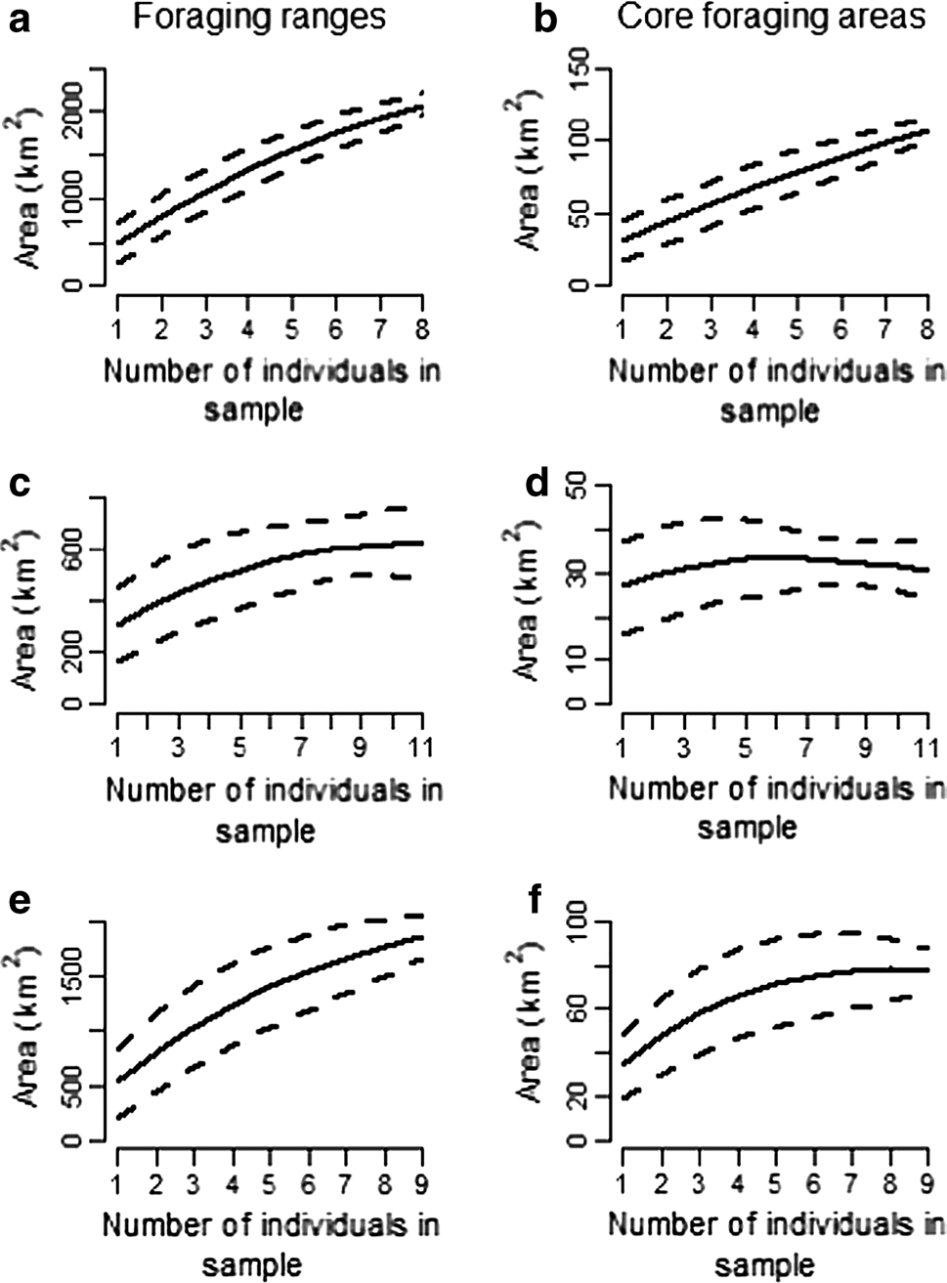
Comparisons of total area (km^2^) covered by foraging ranges and core foraging areas estimated using random samples of 1–8 individuals (incubation 2012), 1–11 individuals (chick-rearing 2012) and 1–9 individuals (chick-rearing 2011) for incubation (a, b), chick-rearing 2012 (c, d) and chick-rearing 2011 (e, f). Curved lines were fitted using a loess spline where α = 1.0. Standard deviations are represented by dashed lines

Kernel density plots illustrated clear differences in the extent of foraging ranges at different breeding stages with birds covering a larger area during incubation in 2012 than during chick-rearing in both years (Incubation = 2219.37 km^2^, *N* = 9 birds, 964 foraging locations; Chick-rearing in 2012 = 678.48 km^2^, *N* = 12 birds, 1539 foraging locations; Chick-rearing in 2011 = 1962.48 km^2^, *N* = 10 birds, 966 foraging locations; Fig. 2). The core foraging area was smaller during chick-rearing in 2012 than during incubation in the same year (Incubation = 116.91 km^2^, Chick-rearing in 2012 = 32.20 km^2^) and showed no overlap between stages (Fig. 3). Core foraging areas during chick-rearing in both years showed an overlap of 17.89 %, and there was a slightly greater degree of overlap in foraging ranges (18.16 %; Fig. 3). Both foraging ranges and core foraging areas during chick-rearing in 2012 were more restricted than those of chick-rearing in 2011 (Foraging ranges: Chick-rearing in 2012 = 678.48 km^2^, Chick-rearing in 2011 = 1962.48 km^2^; Core foraging areas: Chick-rearing in 2012 = 32.20 km^2^, Chick-rearing in 2011 = 78.74 km^2^). Over half of foraging ranges and core foraging areas of birds foraging during chick-rearing in 2012 were found within those of birds foraging at the same stage in the previous year (Foraging range = 70.70 %, Core foraging area = 61.65 %). Area saturation curves showed that chick-rearing 2012 foraging ranges and core foraging areas and chick-rearing 2011 core foraging areas reached asymptote (Fig. 4c, d, f), while those of incubation and chick-rearing 2011 foraging ranges did not. However, increase in foraging area size slowed down as more individuals were included in the sample.

### Prey type and adult body condition

A total of 17 regurgitate samples containing 288 vertebrae were collected over the 2012 breeding season (Incubation: adults *N* = 8, vertebrae = 187; Chick-rearing: adults *N* = 3, chicks *N* = 6, vertebrae = 101). In 2012, 94.1 % (*N* = 16) of samples contained only sandeel (*Ammodytes* spp) vertebrae; the remaining sample from an adult during incubation contained vertebrae from sprat (*Sprattus sprattus*). Regurgitates collected during incubation in 2012 contained larger fish than those collected during chick-rearing in the same year (Incubation: 165.14 ± 16.70 mm, Chick-rearing: 60.78 ± 43.04 mm; LMM (with sample ID as a random factor): χ_1_^2^ = 10.31, *P* = 0.001, *N* = 288). Productivity of the whole colony (number of chicks fledged/nest) was slightly higher in 2011 than in 2012 (1.5 and 1.2, respectively from a range of 0.4–2.0 on Coquet Island from 1991 to 2012 (RSPB unpubl. data)). This inter-annual difference did not coincide with variation in body condition. No significant difference in adult body condition index was found between birds foraging during chick-rearing 2012 and 2011 (1.15 ± 0.03 and 1.15 ± 0.02 g mm^−1,^ respectively; *t*_*27.01*_ = 0.02, *P* = 0.98, *N* = 30). The body condition index of birds foraging during incubation was significantly higher than that of birds foraging during chick-rearing in 2012 (1.26 ± 0.02 and 1.15 ± 0.03 g mm^−1^, respectively; *t*_*28.80*_ = −3.34, *P* = 0.002, *N* = 32).

### Environmental determinants of foraging areas

Birds foraged further from the colony during incubation than during chick-rearing in 2012 (χ_1_^2^ = 41.51, *P* < 0.001, *N* = 220; Table 2) and while there were some similarities in environmental parameters associated with foraging locations between breeding stages, some environmental parameters associated with foraging locations differed. During incubation, individuals foraged in areas of high chlorophyll *a* concentration, low SST and in areas where chlorophyll *a* concentration had been low the previous winter and in the previous month (Table 3), while during chick-rearing in 2012, individuals foraged in areas of low SST (χ_1_^2^ = 102.98, *P* < 0.001, *N* = 120) and winter chlorophyll *a* concentration (χ_1_^2^ = 5.08, *P* = 0.02). Birds foraged further from the colony during chick-rearing in 2011 than in 2012 (χ_1_^2^ = 19.56, *P* < 0.001, *N* = 220; Table 4). We found no effect of date on foraging range during the chick-rearing period in 2012 (χ_1_^2^ = 1.04, *P* = 0.31, *N* = 120) or between chick-rearing in 2011 and 2012 (χ_1_^2^ = 0.89, *P* = 0.34, *N* = 220). Environmental conditions associated with foraging locations were not consistent between years. During chick-rearing in 2012, birds foraged in areas of low SST and winter chlorophyll *a* concentration, while during chick-rearing in 2011, foraging locations were associated with low SST (Table 5), areas of higher winter chlorophyll *a* concentration (χ_1_^2^ = 19.63, *P* < 0.001, *N* = 100) and areas of shallow water (Table 5). There was a significant interaction between SST and bathymetry (χ_1_^2^ = 18.24, *P* < 0.001; Table 5) explaining probability of foraging during chick-rearing in 2011, which suggests that the relationship between foraging locations and SST varied at different water depths. Correlated explanatory variables were excluded from models: bathymetry, SST lag 1 and chlorophyll *a* concentration lag 1 from the chick-rearing 2012 model and SST lag 1 from the chick-rearing 2011 model. This is unlikely to have caused the inconsistency in environmental variables observed between breeding stages and years, as including these variables gave qualitatively similar model results.

**Table 2:** Output from minimum adequate binomial model fitted using REML examining whether birds foraged further from the colony during incubation than during chick-rearing in 2012

	Estimate ± SE	*z* value	*P* value
Intercept	4.17 ± 0.85	4.93	<0.001
Distance from colony	−0.13 ± 0.03	−5.02	<0.001
Stage:			
Chick-rearing	0		–
Incubation	−3.08 ± 0.98	−3.13	0.002
Distance from colony x Stage:			
Chick-rearing	0	–	–
Incubation	0.11 ± 0.03	4.29	<0.001

Random factor = Bird ID. *N* = 210. ROC curve showed the model to fit the data satisfactorily (AUC = 0.88)

**Table 3:** Output from minimum adequate binomial model fitted using REML examining environmental variables associated with foraging locations during incubation 2012

	Estimate ± SE	*z* value	*P* value
Intercept	19.81 ± 7.16	−2.77	0.006
SST lag 0	−2.11 ± 0.78	−2.72	0.006
Chlorophyll lag 0	0.54 ± 0.17	3.13	<0.002
Chlorophyll winter	−0.84 ± 0.42	−2.01	0.04
Chlorophyll lag 1	−0.46 ± 0.24	−1.94	0.05

Random factor = Bird ID. *N* = 90. ROC curve showed the model to fit the data satisfactorily (AUC = 0.84)

**Table 4:** Output from minimum adequate binomial model fitted using REML examining whether birds foraged further from the colony during chick-rearing in 2011 than during chick-rearing in 2012

	Estimate ± SE	*z* value	*P* value
Intercept	1.86 ± 0.48	3.86	<0.001
Distance from colony	−0.05 ± 0.01	−4.37	<0.001
Year:			
Chick-rearing 2011	0	–	–
Chick-rearing 2012	2.10 ± 0.89	2.37	0.02
Distance from colony x Year:			
Chick-rearing 2011	0	–	–
Chick-rearing 2012	−0.10 ± 0.03	−3.64	<0.001

Random factor = Bird ID. *N* = 220. ROC curve showed the model to fit the data satisfactorily (AUC = 0.91)

**Table 5:** Output from minimum adequate binomial model fitted using REML examining environmental variables associated with foraging locations during chick-rearing in 2011

	Estimate ± SE	*z* value	*P* value
Intercept	144.09 ± 32.92	4.38	<0.001
SST lag 0	−14.49 ± 3.26	3.26	<0.001
Chlorophyll winter	1.16 ± 0.32	3.66	<0.001
Bathymetry	2.05 ± 0.50	4.13	<0.001
SST lag 0 × Bathymetry	−0.20 ± 0.05	−4.18	<0.001

Random factor = Bird ID. *N* = 100. ROC curve showed the model to fit the data satisfactorily (AUC = 0.85)

## Discussion

To protect at-sea foraging areas over an appropriate time scale, temporal changes in foraging behaviour must be considered if important areas are to be fully captured. Previous studies have identified foraging areas used by a range of seabird species including kittiwakes from tracking data collected during only one breeding stage (Kotzerka et al. 2010; Stauss et al. 2012; Chivers et al. 2013) or year (Weimerskirch et al. 2005; Votier et al. 2010). Using tracking data over restricted time periods to recommend suitable locations for long-term MPAs are likely to result in seabird foraging areas being underrepresented. Our findings show that foraging areas can change significantly within the breeding season and between years and that environmental variables associated with foraging locations also change over time.

The kittiwake colony on Coquet Island comprised 215 breeding pairs in 2012, which is typical of a smaller colony in the UK where the median colony size is 301 pairs (data from http://jncc.defra.gov.uk/page-4460). Intra-specific competition for food may regulate seabird foraging behaviour (Hunt et al. 1986; Lewis et al. 2001b; Grémillet et al. 2004), with individuals breeding in smaller colonies having shorter foraging ranges than those from large colonies. While such relationships have not been demonstrated for kittiwakes in the UK, it is possible that birds from larger colonies may range more widely than the birds tracked here. The effect of breeding stage on foraging range of kittiwakes nesting in larger colonies is unknown, although one study has examined inter-annual variation in foraging range at larger colonies than that on Coquet Island (Chivers et al. 2013).

Foraging areas exhibited very little overlap between breeding stages. Important foraging areas were situated further to the north of the colony during incubation, while birds foraged close to the colony during chick-rearing in 2012, to the west and south. During incubation, birds made longer foraging trips further from the colony presumably to areas with more predictable resources compared with during chick-rearing when birds made shorter trips to areas closer to the colony. Studies on a variety of different species have found a similar effect with birds foraging further from the colony during incubation than during chick-rearing (Cairns 1987 (common murres); Weimerskirch et al. 1993 (wandering albatross); Jouventin et al. 1994 (king penguins)). Chicks require regular food provisioning shortly after hatching (Weimerskirch et al. 1993; Suryan et al. 2002), and chick demand for food may explain the reduction in trip length we observed during early chick-rearing compared with the incubation period, when adults were less restricted (Weimerskirch et al. 1993; Ojowski et al. 2001). Although studies have shown that adults respond to changing chick demands by varying diet and foraging areas (Williams and Rothery 1990; Robertson et al. 2014), these changes may also be facilitated by changes in food availability over time (Uttley et al. 1994; Myksvoll et al. 2013).

More foraging trips contained overnight components during incubation than during chick-rearing in 2012. Birds may have been less restricted to foraging close to the colony during incubation than during chick-rearing (Weimerskirch et al. 1993; Ojowski et al. 2001), which may have allowed them to undertake longer trips, requiring overnight resting periods, and to exploit distant foraging areas.

While there were some similarities in environmental variables explaining variation in foraging locations between breeding stages, our results suggest that the importance of specific environmental variables linked to foraging change throughout the breeding season. During incubation, birds foraged in areas of higher chlorophyll *a* concentration, while during chick-rearing in 2012, chlorophyll *a* concentration had no effect on foraging location and birds foraged in areas of lower SST and where chlorophyll *a* concentration had been low the previous winter. Sandeel have been shown to aggregate in areas of high chlorophyll *a* concentration (Eliasen et al. 2011), and lower SST has been correlated with increased sandeel recruitment and growth (Arnott and Ruxton 2002; Frederiksen et al. 2004, 2011).

North Sea kittiwakes feed almost exclusively on sandeel during the breeding season (Harris and Wanless 1997; Lewis et al. 2001a; Coulson 2011), but change their feeding habits according to breeding stage. During incubation in May, adults concentrate on older sandeel (1 + year group) to feed themselves and switch to juvenile sandeel (0 year group) to feed both themselves and their chicks during the chick-rearing period in June and July (Wright 1996; Harris and Wanless 1997; Lewis et al. 2001a). Kittiwake breeding success has been shown to correlate with abundance of both 0 group and 1 + group sandeel in the North Sea (Harris and Wanless 1990, 1997; Rindorf et al. 2000), which suggests that both these age classes are necessary for successful reproduction. We found significantly larger (and therefore older) sandeel in adult regurgitates during incubation in May 2012 and smaller sandeel in both adult and chick regurgitates during chick-rearing in June 2012. Juvenile sandeel are readily available in surface waters in June, while older sandeel start to move deeper into the water column at this time (Rindorf et al. 2000), hence temporal changes in diet may reflect variation in abundance of different sandeel age classes (Montevecchi and Myers 1996; Coulson 2011).

While birds travelled further from the colony during incubation, the size-corrected mass of birds tracked at this breeding stage was higher than that of birds tracked during chick-rearing in 2012. Previous studies have shown that adult body mass declines during chick-rearing as birds must work harder to supply both themselves and their chicks with enough food (Weimerskirch 1990; Tveraa et al. 1998; Lormée et al. 2003). Adults can compensate for weight loss during chick-rearing by accumulating fat reserves during incubation and initially feed on large energy-rich prey before switching to smaller prey items to feed chicks (Kitaysky et al. 1999). Birds in our study may have targeted large prey items to accumulate fat reserves prior to chicks hatching when they had to increase their energy expenditure, although it has been suggested that weight loss during chick-rearing is a deliberate strategy by adults to improve flight efficiency (Croll et al. 1991).

Environmental variables such as SST and chlorophyll *a* concentration can change significantly over the course of the breeding season (Pingree et al. 1975; Sharples et al. 2001; Hyrenbach et al. 2002; Peck et al. 2004). Such changes have the potential to affect the distribution and abundance of sandeel of different age classes. Zero group sandeel are smaller than older age classes and are therefore more vulnerable to predation and cannibalism (Arnott and Ruxton 2002). They also have higher metabolic rates and are differentially affected by physical features such as ocean currents, upwellings and temperature (Hayward 1997; Hollowed et al. 2001). Sandeel in the North Sea mainly prey on *Calanus* species, the abundance and distribution of which also depends on oceanographic conditions (Mackas et al. 2001). Prey preference and habitat selection vary among fish of different age classes (Werner and Gilliam 1984), hence 0 group sandeel may utilise different feeding areas to 1 + group sandeel. As kittiwakes in our study exploited sandeels of different age classes between breeding stages, variation in habitat preference (e.g. sediment size) among sandeel age classes may explain differences in environmental variables associated with kittiwake foraging locations we observed during incubation and chick-rearing (Wright et al. 2000; Holland et al. 2005).

We show that foraging areas of birds breeding at the same colony can change significantly during chick-rearing in two consecutive years, confirming the results of previous studies (Wanless et al. 1991; Suryan et al. 2000; Chivers et al. 2012). A study comparing kittiwake foraging behaviour in years of varying food availability showed that trip length and duration increased in years of low food availability resulting in decreased breeding success (Chivers et al. 2012). Both foraging range and core foraging area were larger during chick-rearing in 2011 than in 2012 and birds were more likely to forage further from the colony during chick-rearing in 2011. While there was limited overlap in foraging areas between years, over half of the chick-rearing 2012 foraging range and core foraging area were found within those of chick-rearing 2011. Hence, although birds foraged further from the colony in 2011, birds in both years shared some important foraging areas. Time of day trips were carried out affected duration and maximum foraging range during chick-rearing in both years. However, the percentage of trips that took place at night was similar during chick-rearing in 2011 and 2012 (29.0 % and 22.0 %, respectively); hence, this is unlikely to explain inter-annual variation in trip parameters.

While tracking dates did not overlap between the 2 years (birds were tracked from 14th–17th June 2011 and from 17th June–3rd July 2012), we found no effect of date on the distance birds foraged from the colony between years. Therefore, the difference in foraging range between chick-rearing in 2011 and 2012 is very unlikely to result from seasonal effects. Our analysis shows that birds foraged in areas associated with different environmental variables during chick-rearing in consecutive years. In 2012, birds foraged in areas of lower SST and areas where winter chlorophyll *a* concentrations had been low. While SST and winter chlorophyll *a* concentrations were also significant in 2011, birds were found to forage in areas of deeper water, and winter chlorophyll *a* concentration was shown to have the opposite effect on probability of foraging than during chick-rearing in 2012. These changes may reflect differences in oceanographic conditions between years affecting prey abundance and distribution. In 2012, conditions close to the colony appear to have supported a high abundance of small sandeel, while models and kernel density plots suggest that prey was distributed in patches of productive areas further from the colony in 2011. Productivity of the whole colony was relatively high in 2011 and 2012, suggesting that adequate prey was available in both years (Chivers et al. 2012). Size-corrected mass measurements taken from adults during chick-rearing in both years suggest that adult condition was similar during chick-rearing in 2011 and 2012. Hence, while prey distribution may have differed between the 2 years, there is no evidence to suggest that low food availability affected foraging locations of birds in 2011. Previous studies have shown that foraging behaviour of species breeding at the same colony varies between years (Cairns 1987; Myksvoll et al. 2013) making it necessary to undertake tracking studies over several years of differing food availability to identify useful foraging areas.

Area saturation curves showed that number of individuals included in kernel density estimations affected the estimated size of foraging areas. As curves for foraging ranges and core foraging areas reached asymptote for chick-rearing 2012, this suggests that an adequate number of birds were tracked to accurately represent foraging areas for the whole colony during this breeding stage. However, foraging ranges during incubation 2012 and chick-rearing 2011 did not reach asymptote hence differences in extent of foraging areas used by the whole colony between breeding stages and years may have been even larger area than estimated by this study (Soanes et al. 2013).

The protection of foraging areas to enhance the prey resources on which seabirds depend for successful reproduction would be expected to result in higher levels of breeding productivity. Previous studies have attempted to estimate probable seabird foraging areas using correlations between known foraging locations and associated oceanographic features (Huettmann and Diamond 2001; Nur et al. 2011; Grecian et al. 2012; Lascelles et al. 2012). Prey aggregations for seabirds occur where oceanographic features combine to enhance phytoplankton abundance and hence zooplankton and fish availability, or where currents force prey species to aggregate (Hunt et al. 1999). Features such as chlorophyll *a* concentration and SST vary spatially and temporally (Hunt et al. 1999; Hyrenbach et al. 2000), affecting the location of potential foraging areas. Our study shows how oceanographic features associated with foraging areas vary throughout the breeding season and between years. This has significant implications for the designation of potential MPAs based on habitat suitability as the usefulness of specific areas for foraging will change over time. To designate useful long-term MPAs for seabirds, temporal changes in foraging areas and variation in preference for oceanographic features must be considered. The UK Government is a signatory to international agreements including the EU Birds Directive, Convention on Biological Diversity and the OSPAR Convention whose aims include establishing a network of MPAs and Special Protection Areas (SPAs) incorporating foraging areas used by seabirds, seaducks, waders and divers (Stroud et al. 2001; www.jncc.gov.uk/page-4549). This network is unlikely to adequately represent a significant proportion of seabird foraging areas, as areas useful for foraging are highly variable. The development of dynamic MPAs that vary depending on breeding stage and the location of optimal foraging habitat would complement current proposed sites (Game et al. 2009).

It is becoming increasingly apparent that protection of seabird foraging areas is necessary to prevent population declines brought about by a decrease in food availability. Examining foraging behaviour throughout the breeding season and in more than 1 year results in the identification of larger potential foraging areas than by examining foraging behaviour only during a single breeding stage or year. Our study emphasises the importance of carrying out seabird tracking and examining associated environmental variables during extended time periods when attempting to identify sites for designation as MPAs for seabirds.
